# A qualitative exploration of the implementation facilitators and challenges of a community health worker program to address health disparities for people recently released from incarceration using the consolidated framework for implementation research

**DOI:** 10.1186/s43058-024-00653-1

**Published:** 2024-10-18

**Authors:** Quinn O. Hood, Natalia Irvine, Krina Shah, Shahmir H. Ali, Tamar Adjoian Mezzacca, Michael Serrano, Lorna E. Thorpe, Terry T. K. Huang, Maria R. Khan, Nadia Islam

**Affiliations:** 1 New York City Department of Health and Mental Hygiene, Bureau of Health Promotion for Justice-Impacted Populations, Queens, NY USA; 2 Department of Population Health, New York University Grossman School of Medicine, NYU-CUNY Prevention Research Center, New York, NY USA; 3 Center for Systems and Community Design, Graduate School of Public Health and Health Policy, City University of New York, NYU-CUNY Prevention Research Center, New York, NY USA

**Keywords:** Reentry, People with justice involvement (PWJI), Incarceration, Community health workers, Primary health care, Social services, Health equity, Community-clinical linkage, Structural racism

## Abstract

**Background:**

Despite the potential for community health worker (CHW)-led programs to improve the health of people with justice involvement (PWJI), little is known about the practical implementation of such models. We explored barriers and facilitators to implementation of a municipal CHW program, the Health Justice Network (NYC HJN), led by the New York City Department of Health and Mental Hygiene (DOHMH) in partnership with three reentry-focused community-based organizations (CBOs) and three federally qualified health centers (FQHCs) that was designed to serve the health and social service needs of PWJI.

**Methods:**

Eighteen in-depth qualitative interviews were conducted with a purposive sample of CHWs, partner site supervisors, and DOHMH staff. Interviews were conducted virtually and transcribed verbatim. Codes and themes were developed using the Consolidated Framework for Implementation Research (CFIR) to understand facilitators and barriers to NYC HJN implementation.

**Results:**

Important facilitators to implementation included: lived experience of CHWs, as well as NYC HJN’s voluntary nature, lack of onerous eligibility criteria, and prioritization of participant needs. One barrier was the lack of a formal implementation protocol. Themes identified as facilitators in some instances and barriers in others were CHW integration into site partners, the expansive scope of work for CHWs, the integration of a trauma-informed approach, values alignment and existing infrastructure, leadership engagement, CHW training and support, and input, feedback, and communication.

**Conclusions:**

Findings will help inform how to successfully implement future CHW-led interventions for PWJI with municipal, health, and social service partners.

**Supplementary Information:**

The online version contains supplementary material available at 10.1186/s43058-024-00653-1.

Contributions to the literature

- There is a dearth of knowledge regarding local municipal health department community health worker led programs to improve the health and well-being of individuals with criminal justice involvement. Understanding the intricacies of the practical implementation of the co-located community health worker led program can ameliorate health disparities.
- We found facilitators and barriers that underpinned the program’s implementation process, giving insights on the replicable nature of the program that can be enacted in various jurisdictions.
- Our findings emphasize the gaps in literature required for future successful implementation using these approaches across community and clinical settings.

## Background

The United States has the largest population under correctional supervision in the world, with nearly 2 million people incarcerated [1]. Following release from incarceration, people with justice involvement (PWJI) face multiple impediments to medical care [2]. In the first 12 months after release, the mortality rate among PWJI is 3.5 times that of the general population [3]. PWJI suffer from adverse health outcomes including traumatic brain injury [4], cardiovascular disease [5], hypertension [6], diabetes [7], hepatitis C [8], and HIV [9], among others. Factors such as poverty, prior trauma, psychiatric symptoms, and co-occurring substance use predate incarceration and can exacerbate common health risks [10–16]. Psychosocial factors associated with incarceration, including the criminal legal process [17], isolation and stigma [18], also negatively impact PWJI. Additionally, PWJI face disparate rates of mental health and substance use disorders [19].

For many PWJI, the post-release and community reentry process is a stressful experience due to the need to re-establish social and healthcare needs that have been disrupted [20–23]. The navigation of these services play a crucial role in improving the overall health of PWJI. Trauma-informed services are an important way to address the burden of trauma on health in primary care and community settings [24, 25] and are especially critical for PWJI, but significant gaps remain in understanding how to best implement and scale these approaches.

One promising model developed to address the health needs of PWJI is a community health worker (CHW)-facilitated primary care program. The first such program was the Transitions Clinic Network model (TCN), launched in San Francisco in 2006 [26]. It was designed with input from formerly incarcerated people who had been released without connections to care. The TCN hired and trained CHWs with lived experience, integrating them into existing primary care systems. Research has shown that TCN participants had fewer emergency department visits, lower odds of returning to prison for a violation of parole or probation, and lower criminal legal system cost than PWJI who did not participate in the program [27]. While there is evidence on the value that CHWs bring to health and reentry services for PWJI [28], little is known about barriers and facilitators of such programs that determine successful implementation of this model.

### The New York City Health Justice Network

Leveraging the TCN and other models, in 2019 the NYC Department of Health and Mental Hygiene (DOHMH) launched the NYC Health Justice Network (NYC HJN) to address barriers and health inequities experienced by people returning to the community from incarceration, with funding from the Manhattan District Attorney’s Office Criminal Justice Investment Initiative [29].

The NYC HJN program is a CHW-facilitated community-clinical linkage to care program. The program consists of a partnership of three federally qualified health centers (FQHCs) and three CBOs, utilizing a trauma-informed approach.

The NYC HJN program is overseen and coordinated by DOHMH, with CHWs with lived experience of the criminal legal system employed, hired, and embedded within partner sites.. Mirroring populations within the New York State prison system and NYC jail system [30, 31], participants in the NYC HJN program are mostly male (90%), and Black (50%) or Hispanic/Latino/a (28%). The CHWs help connect participants to programs and services tailored to their needs such as primary care, acquiring vital documents, job training, employment, benefits, housing, food assistance, substance use treatment, behavioral health treatment, and education.

The NYC HJN program is voluntary and open to anyone 18 or older who has been released from jail or prison within the last three years. The program is participant-centered, giving participants the ability to drive their own goals and needs. From September 2019 through December 2023, HJN served over 1,300 unique participants.

### Study aim

The purpose of this investigation was to examine the barriers and facilitators to the implementation of the NYC HJN program to inform future replication and scale-up efforts. Findings from this study can inform future partnerships and programs directed at improving the health and well-being of PWJI, in addition to offering insight into how best to structure partnerships between local health departments, CBOs and FQHCs.

## Methods

### Implementation science theoretical framework

The Consolidated Framework for Implementation Research (CFIR) informed data collection and analysis for this study [32], CFIR was chosen for its capacity to comprehensively analyze barriers and facilitators of an intervention and compare these with implementation analyses of similar interventions. The framework is comprised of five domains of the intervention: intervention characteristics (e.g., the adaptability, complexity, and quality), characteristics of individuals (e.g., the beliefs, circumstances, and self-efficacy of individuals), inner setting (e.g., the internal dynamics between those implementing), outer setting (e.g., relationships with external stakeholders and the role of the intervention in the broader community), and process (e.g., meta-level considerations on how the intervention was planned, executed, and evaluated).

### Sample/recruitment

The study sample consisted of a purposive sample of 18 key informants who were directly involved in the planning, implementation, and/or delivery of the NYC HJN program. The research team agreed not to include HJN participants in the sample at DOHMH’s request and to ensure participant privacy. Since the research team acted as external independent evaluators, they relied on the DOHMH team to provide them with a list of key informants that were involved in the implementation of the program. With the permission of DOHMH, study participants were emailed directly with a description of the study and its purpose. Study team members worked with participants to identify a convenient time for the interview. In total, six DOHMH staff members were contacted and interviewed. The study team attempted to contact nine partner site leaders but were able to reach and interview seven due to staff turnover. The study team was unable to locate contact information for the two staff members who were no longer employed at the partner sites. Of the six CHWs that were contacted, five agreed to participate and were interviewed. Interviews lasted from 38 to 82 min and were conducted from May through September 2022 by five different interviewers with qualitative research training. All interviews took place over Zoom, were audio-recorded with the participant’s consent, and transcribed using GMR Transcription [33]. The study was approved by the Institutional Review Boards at the NYU Grossman School of Medicine and DOHMH.

### Data collection

Three different semi-structured interview guides representing each of the three key stakeholder groups were developed and tailored to address the CFIR framework domains of intervention characteristics, inner setting, outer setting, and characteristics of individuals. Within these four domains, all three interview guides had questions about complexity, relative advantage, design quality and packaging, compatibility, cosmopolitanism, networks and communication, available resources, and learning climate constructs. Interviews were only conducted approximately three years after the implementation of the program, and not at baseline, therefore making it impossible to include questions related to the process domain in the interview guides. All interviewers were trained in interviewing and coding with the developed study guides by a senior researcher with expertise in qualitative research Table 1.

**Table 1:** Relevant CFIR domains, constructs, and themes

Domain	Domain Description	Constructs	Themes
Intervention Characteristics	Covers the key aspects of an intervention that influence the success of implementation	- Adaptability - Complexity - Costs - Design quality and packaging - Evidence strength and quality - Intervention source - Relative advantage - Trialability	Facilitator: - Lived experience of CHWs with justice involvement (complexity and relative advantage) - Lack of restrictions to enrollment and voluntary nature of services (design quality and packaging) Facilitator and Challenge: - CHW integration into CBOs and healthcare settings (complexity and adaptability) - Expansive scope of work for CHWs (complexity and relative advantage) - Integration of trauma-informed approach (design quality and packaging)
Inner Setting	Refers to the setting within which the intervention was implemented. In the case of NYC HJN this includes both DOHMH and the six partner sites where CHWs were embedded	- Structural characteristics - Networks and communications - Culture - Implementation climate - Readiness for implementation	Barrier: - Lack of a formal implementation protocol (access to knowledge and information) Facilitator and Challenge: - Values alignment and existing infrastructure (compatibility and culture, relative priority and available resources) - Leadership engagement (leadership engagement) - CHW training and support (learning climate and networks and communication) - Input, feedback and communication (networks and communication)
Outer Setting	Encompasses external influences on program implementation. In the case of NYC HJN, this was anything outside of DOHMH and the six partner sites	- Cosmopolitanism - External policies and incentives - Patient needs and resources - Peer pressure	Facilitator: - Prioritizing participant needs (patient needs and resources, cosmopolitanism, and external policies and incentives)

### Data analysis

All interviews were recorded and transcribed prior to analysis. The first author had extensive prior experience of conduction research about the criminal legal system. Analyses were conducted using a framework approach which involved deductively applying the CFIR framework to generate insights related to each construct, while also generating some additional inductively derived themes from the qualitative data relevant to each construct. Specifically, an initial basic codebook was first developed by SA using four of the five CFIR domains. The analysis team then independently applied the initial codebook to a set of five transcripts; coded transcripts were reviewed as a group to revise and generate additional codes tailored to findings from the interviews. The revised codebook was then applied by coders to analyze the remaining transcripts; each coded transcript was independently reviewed by a second coder, with discrepancies addressed through mutual consultation (and if needed, third coder). Upon completion of coding, salient themes inductively generated from the qualitative data classified within each CFIR constructs were then identified and presented in the report.. The analysis was conducted by three graduate-level students and two graduate-trained staff with prior qualitative research training, supervised by NI. Analysis was conducted using the open-source qualitative coding software Taguette [34].

## Results

### Intervention characteristics

#### Lived experience of CHWs

Among staff members, a consistently identified facilitator of program success was the importance of employing CHWs who had lived experience of the criminal legal system. CHWs discussed not just the knowledge they had of the reentry process but also how their awareness of going through that process themselves enabled patience and empathy with participants experiencing a range of challenging situations.

“I think if we had CHWs who didn’t have lived experience, that it would have been a lot more difficult to engage with our participants. That’s something that I can directly draw from. And when they have any challenges, without giving them too much information and say, ‘Hey, I remember when I experienced that too during my transition, and this is what helped me.’” (CHW)

#### Lack of restrictions to enrollment and services

No one was excluded from enrolling in NYC HJN due to conviction history or any other criteria. This was frequently cited as a key component of the program and what made it stand out in relation to existing social programs for PWJI.

“The services are phenomenal. I think ‘cause we were open to services, we serve anybody regardless of their crime. So, I think that’s huge. I think some populations are not welcome in different programs…. It was voluntary, which was great. So, most of the people that signed up, their probation office, or parole officer weren’t making them sign up; they signed up because they wanted to make some change in their lives.” (DOHMH)

#### CHW integration into CBOs and healthcare providers

The CHWs’ ability to connect participants to services was facilitated by embedding them at partner organizations, allowing for referrals to be made more seamlessly.

“All our programs always ask for peer support and community health worker support to help people get the services that traditional billing mechanisms aren’t paying for…. Staff had really wanted this for a long time. So, as soon as they were told it was available, they’re like, ‘Yes, we have participants. Please help them’.” (Site)

Although embedding CHWs in this way was a key feature of NYC HJN, it sometimes created role confusion for CHWs between DOHMH and the sites.

“I think there was some issues with us trying to be on the same page as a community organization. The issue is when you work with other organizations, they have different deliverables.” (DOHMH)

#### Expansive CHW scope of work

CHWs were dedicated to serving the participant needs, no matter how difficult it was to find a particular service or provider. CHWs often went above and beyond for participants.

“I would look for a community-based organization within certain areas or neighborhoods and I'd reach out to them. So, if certain places do maybe vocational trainings, then I'd call and inquire and ask about what kind of different services they provide and what are the eligibility requirements and so on and so forth….I would stop by and try to pick up conversations with the people, with whatever organization they're from, and try to get an idea of what they do and see if I can leverage those organizations to deliver services to the participants.” (CHW)

Despite the lengths that CHWs went to meet needs, some staff members discussed the challenge of not having a list of resources within NYC HJN and the great amount of work required to maintain their own networks, relying on each other for connections. “Resource mining” was one of many skills required to be a successful CHW.

“Something that I didn't foresee when we were hiring and integrating folks in the site is that our CHWs were doing such an incredible amount of work and were relied on in so many different ways that not one human being could ever have the capacity and competency in that area.” (DOHMH)

#### Integration of trauma-informed approach

One of the innovative components of NYC HJN was the incorporation of a trauma-informed approach. According to a stakeholder from DOHMH:

“We took a two-prong approach. One was to work with those that were delivering the services, and we envision that being folks’ kind of across different disciplines on skills and tools that can support themselves while they’re delivering the services as well as skills and tools they could teach participants, clients, and patients. So, that was the social resilience model, train-the-trainer model. And then, the second prong was to work on an organizational level in coming in to talk with leadership and folks across different disciplines integrating trauma and resilience practices into the organization. The idea was to create blueprints for change in which leadership would identify areas that they could commit to into integrating that into their culture.” (DOHMH)

Some of the site partners described how NYC HJN’s focus on trauma-informed care helped push their organization to incorporate those principles:

“When I started at the organization, there was no formal trainings for trauma-informed care until the HJN programming started, and that collaboration started. At that point, they decided to make this an organizational effort to have everyone trained in trauma-informed care and now we have a trauma-informed care team who maintains trainings for all the sites.” (Site)

Many NYC HJN stakeholders talked about the importance of designing the program to be trauma-informed; CHWs also mentioned their attempts to be trauma-informed in their interaction with clients.

“So, just trying to make sure that as far as being trauma-informed, if anything that I remember feeling during my reentry that felt uncomfortable to me, making sure – I’m trying to make sure that I wasn’t retriggering anybody during their experience with me, whether that be again, not telling them, ‘You need to do X, Y and Z,’ and having them feel like I’m their parole officer or their probation officer.” (CHW)

Although many stakeholders praised the trauma-informed aspect of the program, some staff interviewed reported that trainings were not successfully implemented across all sites.

### Inner setting

#### Lack of implementation protocol

Although all stakeholders noted that CHWs did a remarkable job of supporting participants and connecting them with services, one barrier highlighted by multiple stakeholders was the absence of a protocol to implement NYC HJN. When asked if they received guidelines or a protocol, one site supervisor responded:

“I would say not exactly, I mean, I learned what the kind of standards were when I came on from the person that had been supervising [name redacted] previously, and from [name redacted], and then speaking with the DOH supervising team. I didn’t get a document or anything outlining protocols or anything.” (Site)

One CHW also talked about the need for more protocols and information:

“A lot of it was me – at least, this is what I felt – winging it. I think we did a decent job to be honest, but I did at times feel like I was winging it and trying to figure out and find resources on the fly for some participants here and there.” (CHW)

#### Values alignment and existing infrastructure

One unique aspect of the program is that although it was administered and managed by DOHMH, the program CHWs were embedded at three CBOs and three FQHCs. The success of NYC HJN varied greatly based on the values and culture of the partner site and its alignment with the goals of NYC HJN.

“HJN worked out of my site, which is the [neighborhood] site that I oversee, and one of the things I always ensure is that we have our biweekly team meetings – our [neighborhood] meetings. So – HJN was always involved. We always allowed HJN to share updates of what’s going on” (Site)

The integration of CHWs into the workflow of these organizations was facilitated by incorporating them into already existing departments, such as care management. At some sites, the CHWs’ job was made more challenging because the site did not offer opportunities for integration with organizational services or lacked services for PWJI altogether.

"But aside from the ones that I learned randomly from colleagues I would talk to, we didn't really have too many HJN services, at least to my knowledge, that we – I don't know how to put it. We just didn't have the amount of support in certain ways that I expected when I first joined the job. A lot of it was me – at least, this is what I felt – winging it. I think we did a decent job to be honest, but I did at times feel like I was winging it and trying to figure out and find resources on the fly for some participants here and there. My peers and colleagues have been great support as far as being able to give me what I need. But I think overall HJN as the organization could definitely do better as far as having – a right off the boat new employee can have a list of resources and contacts – names – that they could reach out to directly for certain things.” (CHW)

#### Leadership engagement

The successful integration of CHWs was aided by passionate site partner leadership who were invested in the program and ensured adequate resources were made available at their institutions. Having a dedicated champion at the FQHC sites was an even larger facilitator, because clinical settings lacked experience of working with PWJI, as opposed to the partner CBOs. At some NYC HJN clinical sites, leadership enthusiastically embraced NYC HJN and used the program to expand the knowledge and competencies of their organizations and staff. In fact, some respondents noted that partnership with HJN helped clinical sites better characterize and enumerate PWJI and supported efforts to expand clinical services to better services. Conversely, sites where the supervisor did not have the capacity to engage fully in NYC HJN had more difficulty integrating CHWs.

“There were a couple sites where the site supervisor was so pro-HJN, and very keen, and had supervised CHWs before, and really adopted our CHW as part of their CHW team. It provided them with all the support that everyone else there got. And then, there were other sites where the site supervisor wasn’t really engaged. They had just all been told they had to do this new grant and they had like 1,000 other things to do, and there was a lot of CHW turnover at that site.” (DOHMH)

#### CHW training and support

The NYC HJN program offered a large amount of training opportunities to CHWs. Most stakeholders, including CHWs, talked about the strong benefits of these opportunities.

“We always – have training opportunities. So, I’ve always provided a space for my CHW to share what are some of the things they would like to get trained on and how they can grow within their position in the company. And so, we always have opportunity for trainings and also supervision.” (Site)

Multiple CHWs were able to rise through the ranks to higher positions within NYC HJN and spoke very highly of the opportunities provided. Some expressed frustration with compensation rates for CHWs, given the amount of work required to successfully engage clients. Additionally, some staff members noted that education requirements at DOHMH prevented some PWJI from having access to certain jobs, which created confusion and challenges.

#### Input, feedback, and communication

Several program stakeholders indicated that they felt their feedback was valued and that there was adequate opportunity to provide feedback on how the program was operating. CHWs were consistent in identifying that they had a close relationship with each other and a culture of helping each other out.

“I think HJN gave everybody a space where they could feel comfortable sharing their opinions and their ideas without there being retaliation or feeling uncomfortable or I can’t share with this person because, you know, they’re going to look at me differently. Our team was very eclectic. We were a band that was meant to be put together, and you know, they heard us.” (CHW)

One challenge that was brought to light by DOHMH staff and partner site staff was that CHWs were required to report to two different supervisors, one at their site and one at DOHMH. Site supervisors were frustrated by the lack of information they received on CHWs work from DOHMH, including not having access to the program’s case management system, and expressed a desire for a stronger communication with DOHMH staff. Some site supervisors felt the lack of clear information prevented them from providing the best possible support to CHWs.

“I think it's a model that can work as long as it — there’s clear expectations set…. I found myself more often than not, hearing the information for the first time while my peer was in supervision with us. And I’d have to often say, ‘I have to look into it and get back to you.’” (Site)

### Outer setting

#### Prioritizing participant needs

Overall, the program was able to fulfill the varied and competing needs of NYC HJN participants through the hard work of the CHWs. CHWs were skilled in prioritizing the needs of every NYC HJN participant, and networked relentlessly to find the best possible services.

Beyond working to fulfill the material needs of participants, CHWs also did a lot of work to address the emotional needs that many PWJI have:

“A lot of people who come out of incarceration don’t have anyone. And so, just having someone that can show them the ropes, and that they can count on for help when they’re in a pinch, and that can just tell them how it is. Tell them how they did it when they came home and speak from a place of experience that it is going to get better. It is gonna get easier. It is gonna be worth it. I think it’s really invaluable.” (DOHMH)

### Lack of access to resources

The lack of affordable housing in New York City was repeatedly identified as the most challenging need to meet for participants:

“One of the difficulties that we often find is housing for individuals, and our shelter system in New York City is lacking, for lack of a better word. And unfortunately, those systems aren’t set up for people to be successful.” (Site)

Obtaining vital documents was so crucial to participants’ wellbeing that HJN developed an internal process for streamlining the acquisition of documents:

“We started doing things ourselves like birth certificate applications. We found out kind of a bit into the program that a lot of people were coming out requesting ID ‘cause they leave jail or prison without any ID and they need their ID to get a job, get a driver’s license. So, we had a lot of vital document requests. And so, we started just doing that ourselves. For a while, I was phoning participants and helping them fill out a birth certificate application with them online.” (DOHMH)

Of note, parole officers were not supportive, wanting participants to meet benchmarks of parole but not assisting in that goal. Several CHWs mentioned the need for the NYC DOHMH to be more directly involved with policy work for the population impacted by the criminal legal system.

### CHW networking

The partnerships the HJN program has leveraged have been successful for employees within the program and participants:

“So, we were able, within our network, to service almost all their needs and then reaching out to other organizations in New York City because we didn’t just have to refer to those organizations, our partner organizations, we could refer to any organization. So, I think building those services for our participants to go back to school, all these extra things that weren’t really part of our first thought when implementing the program. We were able to refer.” (DOHMH)

CHWs acknowledged that it wasn’t solely the connections or partnerships of the HJN program or six agencies that kept the program successful, but their personal connections from former work and their personal determination to create those opportunities. Partnerships with employment organizations seemed to be the most successful and maintained.

CHWs, their supervisors, and agencies worked diligently to create new partnerships:

“I think that we do a really good job engaging with community partners. We're having presentations constantly, people coming to talk to us so that we could refer to them, but also us presenting on our program. Referrals go both ways, so we were having a lot of that kind of communication.” (DOHMH)

Although CHWs worked diligently to facilitate connections to services for participants, the lack of institutional support for programming proved challenging at times. The COVID-19 pandemic disrupted several of these new partnerships and CHWs were forced to ‘resource mine’ several times over. New partnership organizations suggested throughout the interviews included the Department of Motor Vehicles, the Social Security Office, clinical partners, education programs, and the New York City municipal identification program (IDNYC). Additionally, having a CHW present in the transitional hotels was identified as a possible successful strategy:

“This is specifically during COVID. They’re released into an actual hotel. So, we have six hotels, and they get their own room. They get their own bathroom. There are some onsite services through a subcontractor, but it’s very disconnected. So, to have a community health worker there on site would just be a natural partnership.” (DOHMH)

Many CHWs reiterated the need for behavioral and mental health professional partners. Lastly, one CHW discussed collaborating with an organization that reaches those currently incarcerated with information regarding release and the HJN program. They mentioned several attempts to do so, but did not succeed due to feeling unwelcome.

## Discussion

One key theme in the outer setting constructs was prioritizing participant needs. These results of NYC HJN point to the importance of programs to help PWJI define their own priorities and work with them to achieve those goals on their own terms. Other studies have confirmed that implementing participant-centered care contributes to better health and social outcomes [35, 36]. Study respondents discussed several intervention characteristics as program facilitators, such as CHWs’ lived experience, simple program eligibility and ease of access, meaningful integration with partner sites, and utilizing a trauma-informed approach. Program integration and value alignment with partner sites are vital for the sustainability of the program. Our study demonstrates that adopting models such as NYC HJN improves linkages between providers, ultimately benefiting the population served [36]. Embedding CHWs at partner sites that had the capacity and resources to incorporate them into the team was crucial Within HJN, training and ongoing support for CHWs were important facilitators to ensure high-quality of services and for professional development. Organizational and staff buy-in and administrative support have been identified as key elements for integrating peer workers, such as CHWs [37].

Our findings complement several studies that have examined the impact of community-based health interventions to enhance the health of PWJI [38, 39]. To our knowledge, our study represents the first to use the CFIR framework to explore the implementation of a CHW-led health-focused intervention for PWJI. By grounding our analysis in the CFIR framework, our study makes a unique contribution to the field of community-clinical linkage interventions to reduce health disparities.

Our findings corroborate with previous research suggesting community-based CHW interventions hold the most potential for addressing the challenges of PWJI; familiarity and identification in community is the strength of the CHW intervention model [40]. A previous CHW-conducted qualitative study demonstrated that having a formerly-incarcerated CHW was crucial for individuals in building connections and trust in our health care system [41], and is potentially the most effective method of health promotion for disadvantaged communities [42].

### Limitations/ strengths

This study had several limitations. First, the data was collected only from people directly involved in implementing the intervention. Other stakeholders not directly involved were not included in the sample, and HJN participants were not interviewed. Since interviews were conducted as the program was still up and running and many of the staff and community members involved in the initiation of the programs were no longer available, we were not able to include an assessment of CFIR “process” constructs during the development and implementation of the program. Additionally, since HJN was implemented in New York City, not all findings may be transferable to other jurisdictions. Despite these limitations, our study makes a significant contribution to the field of implementation science by utilizing the CFIR framework to assess the implementation of a multi-sector and partnered initiative with a broad goal of engaging PWJI in accessing health and social services.

## Conclusion

This study explored the facilitators and barriers to implementing the NYC HJN program. The results highlight the importance of hiring CHWs with lived experience, the lack of restrictions to enrollment, CHW integration, integration of trauma-informed approach, values alignment and existing infrastructure, leadership engagement, CHW training and support, feedback and communication and prioritizing participant needs. Tackling incarceration as a key social determinant and providing strategies to mitigate its effects are essential to advancing health equity. Efforts to enhance, scale, and sustain programs like NYC HJN should be prioritized across municipal, clinical, and social service partners. Incorporating lessons learned from this study, replication of NYC HJN will increase engagement, services, and linkage to social, health, and behavioral health services, with potential to decrease health disparities among PWJI.

## Supplementary Information

Supplementary Material 1.

## Data Availability

The interviews generated and analyzed during the current study are not publicly available to protect the identities of participants.

## Abbreviations

CBO: Community-Based Organization
CFIR: Consolidated Framework for Implementation Research
CHW: Community Health Worker
DOHMH: New York City Department of Health and Mental Hygiene
FQHC: Federally Qualified Health Center
NYC HJN: New York City Health Justice Network
PWJI: Persons with Justice Involvement
TCN: Transitions Clinic Network
